# Breast cancer metastasis suppressor 1 (BRMS1) attenuates TGF-β1-induced breast cancer cell aggressiveness through downregulating HIF-1α expression

**DOI:** 10.1186/s12885-015-1864-y

**Published:** 2015-10-31

**Authors:** Kyung Hwa Cho, Seong-Lan Yu, Do Yeun Cho, Chang Gyo Park, Hoi Young Lee

**Affiliations:** 1 Department of Pharmacology, Myunggok Medical Institute, College of Medicine, Konyang University, Daejeon, Republic of Korea; 2 Department of Hematology & Oncology, Myunggok Medical Institute, College of Medicine, Konyang University, Daejeon, Republic of Korea

**Keywords:** BRMS1, HIF-1α, Snail, TGF-β1, TWIST1

## Abstract

**Background:**

Cancer metastasis is a multi-step event including epithelial-to-mesenchymal transition (EMT). Breast cancer metastasis suppressor 1 (BRMS1) is a novel metastasis suppressor protein without anti-proliferating activity. However, a detailed underlying mechanism by which BRMS1 attenuates cancer cell EMT and invasion remained to be answered. In the present study, we report an additional mechanism by which BRMS1 attenuates Transforming growth factor-beta1 (TGF-β1)-induced breast cancer cell EMT and invasion.

**Methods:**

Experimental analysis involving chromosome immunoprecipitation (ChIP) and luciferase reporter assays were used to validate hypoxia inducible factor-1alpha (HIF-1α) as a transcriptional regulator of TWIST1 and Snail. Quantitative RT-PCR was used to analyze transcript expression. Immunoblotting and immunofluorescence were used to analyze protein expression. Matrigel-coated *in vitro* invasion insert was used to analyze cancer cell invasion.

**Results:**

BRMS1 strongly inhibited TGF-β1-induced breast cancer cell EMT and invasion. Unexpectedly, we observed that BRMS1 downregulates not only TWIST1 but also Snail expression, thereby inhibiting breast cancer cell invasion. In addition, we provide evidence that HIF-1α is required for Snail and TWIST1 expression. Further, BRMS1 reduced TGF-β1-induced HIF-1α transcript expression through inactivation of nuclear factor kappaB (NF-κB).

**Conclusion:**

Collectively, the present study demonstrates a mechanical cascade of BRMS1 suppressing cancer cell invasion through downregulating HIF-1α transcript and consequently reducing Snail and TWIST1 expression.

## Background

Cancer metastasis is a multi-step event including epithelial-to-mesenchymal transition (EMT) [1]. To start invading surround extracellular matrix, tumor cells should be detached from the neighboring epithelial cells by reducing the expression of E-cadherin. Hypoxic condition and various growth factors including transforming growth factor-beta1 (TGF-β1) and epidermal growth factor (EGF) have been shown to induce EMT through upregulating the expression of transcription factors Snail and TWIST1 [2–4].

Breast cancer metastasis suppressor 1 (BRMS1) is a novel metastasis suppressor protein initially identified by differential display to compare highly metastatic breast carcinoma cells with related but metastasis-suppressed cells [5]. BRMS1 is a part of a family that includes suppressor of defective silencing 3 (SUDS3 or mSds3) and BRMS1-like (BRMS1L or p40) [6, 7] and has been shown to selectively suppress metastasis without any inhibition of tumorigenicity of multiple human cancer cells including melanoma [8], ovarian cancer [9] and non-small cell lung cancer [10]. Accumulating evidence suggests two important mechanisms for suppression of BRMS1-induced cancer metastasis: interaction with chromatin remodeling and inhibition of nuclear factor-kappaB (NF-κB) activity [11]. BRMS1 recruits histone deacetylase1 (HDAC1) to NF-κB consensus binding regions [12, 13] and upregulates miR-146a, leading to downregulation of EGFR expression in breast cancer cells [14]. In addition, BRMS1 was reported to have E3 ligase function, leading to suppressing lung metastasis [15].

Recent studies suggest that BRMS1 suppresses breast cancer cell metastasis through modulating cancer cell EMT. Loss of BRMS1 promotes EMT through NF-κB-dependent-TWIST1 expression [16]. Further, Gong *et al*. [17] claimed that BRMS1 epigenetically silences a receptor for Wnt signaling FZD10, leading to suppress breast cancer cell EMT. However, detailed underlying mechanism by which BRMS1 attenuates cancer cell EMT has not been fully characterized. In the present study, we observed that BRMS1 efficiently inhibited TGF-β1-induced breast cancer cell EMT and invasiveness by downregulating not only TWIST1 but also Snail expression. In addition, we provide evidence that NF-κB is implicated in TGF-β1-induced hypoxia inducible factor-1alpha (HIF-1α) expression and subsequent Snail and TWIST1 expression. Further, the present study shows that BRMS1 significantly inhibits TGF-β1-induced HIF-1α transcript, leading to downregulation of Snail and TWIST1. Therefore, our results identify mechanism by which BRMS1 attenuates cancer cell progression through downregulating HIF-1α and subsequently reducing Snail and TWIST1 expression.

## Methods

### Reagents

TGF-β1 was obtained from R&D systems (Minneapolis, MN). BAY11-7082 was purchased from Calbiochem (La Jolla, CA). The pcDNA3-BRMS1 plasmid was generated by subcloning *Eco*RI/*Xho*I from pOTB7-BRMS1 (clone ID: hMU011011, KRIBB, Korea). The empty pcDNA3 vector was used as a negative control.

### Cell culture

All breast cancer cell lines were purchased from American Type Culture Collection (Manassas, VA). MDA-MB-231 were maintained in RPMI 1640 supplemented with 10 % fetal bovine serum (FBS) and 1 % penicillin/streptomycin. MCF-7 cells were maintained in Dulbecco’s modified Eagle’s medium, supplemented with 10 % fetal bovine serum and 1 % penicillin/streptomycin. All cells were incubated at 37 °C under 5 % CO_2_ in a humidified incubator.

### Small interfering RNA (siRNA)

siRNAs of HIF-1α was obtained from Sigma-Aldrich (St. Louis, MO). Control scrambled-siRNA was obtained from Invitrogen (Carlsbad, CA). Transfection was performed by utilizing Lipofectamine RNAiMAX (Invitrogen, Carlsbad, CA) according to the manufacturer’s instructions.

### Quantitative RT-PCR

Total cellular RNA was isolated from cultured cells using Trizol (Invitrogen, Carlsbad, CA), and 1 μg of RNA was reverse transcribed using oligo (dT) and M-MLV reverse transcriptase (Promega, Madison, WI) according to the manufacturer’s protocol. Reactions were performed as described previously [18]. The primer sequences of mutants are shown below. HIF-1α; forward 5’-GTT TAC TAA AGG ACA AGT CAC C-3’ and reverse 5’-TCC TGT TTG TTG AAG GGA G-3’, TWIST1; forward 5’-GTC CGC AGT CTT ACG AGG AG-3’ and reverse 5’-CCA GCT TGA GGG TCT GAA TC-3’, E-cadherin; forward 5’-ACA GCC CCG CCT TAT GAT T-3’ and reverse 5’-TCG GAA CCG CTT CCT TCA-3’, Snail; forward 5’-TTT ACC TTC CAG CAG CCC TA-3’ and reverse 5’-GGA CAG AGT CCC AGA TGA GC-3’, Slug; forward 5’-TCT GCA GAC CCA TTC TGA TG-3’ and reverse 5’-AGC AGC CAG ATT CCT CAT GT-3’, GAPDH; forward 5’-ACA GTC AGC CGC ATC TTC TT-3’ and reverse 5’-ACG ACC AAA TCC GTT GAC TC-3’.

### Immunoblotting

The cell lysates were prepared as described [19]. Antibodies for HIF-1α (1:1000), p-p65 (1:1000), Snail (1:1000) and Slug (1:1000) were from Cell Signaling Technology (Danvers, MA). Antibodies for E-cadherin (1:1000), TWIST1 (1:1000), BRMS1 (1:500), p52 (1:500), p50 (1:1000) and GAPDH (1:3000) were from Santa Cruz Biotechnology Inc. (Santa Cruz, CA). Antibody for p65 (1:1000) was obtained from BD Biosciences (San Jose, CA). Secondary antibodies for anti-rabbit (1:2000 ~ 5000) and anti-mouse (1:2000) were Thermo Fisher Scientific Inc (Rockford, IL). Secondary antibody for anti-goat (1:3000) obtained from Santa Cruz Biotechnology Inc. (Santa Cruz, CA). The immunoreactive bands were visualized by ECL (Thermo Fisher Scientific Inc., Rockford, IL) using ImageQuant 300 (GE Healthcare, Buckinghamshire, UK).

### Luciferase assay

A TWIST1 luciferase reporter vector [3] was kindly provided from Dr. Hung, MC (M.D. Anderson Cancer Center, Houston, TX). A Snail luciferase reporter vector [20] was kindly provided from Dr. Yook, JI (Yonsei University College of Medicine, Korea). A HIF-1α luciferase reporter vector was obtained from Addgene (Cambridge, MA). The cells were prepared in 12-well plates in triplicate and transfected with the indicated reporter plasmids by utilizing Lipofectamine 2000 reagent (Invitrogen, Carlsbad, CA). After stimulation with or without TGF-β1, the cells were washed twice with ice-cold PBS and harvested with a reporter lysis buffer (Promega, Madison, WI). The luciferase activity was analyzed as described previously [21].

### *In vitro* invasion assay

*In vitro* invasion assay was performed with invasion assay kit with Matrigel-coated inserts (BD Biosciences, San Jose, CA) as described previously [22]. Volume of 1 x 10^6^ cells/well was added to the upper compartment of the invasion chamber. To the lower compartment, we added serum-free conditioned medium with or without TGF-β1. After incubation for 12 h (MDA-MB-231) or 48 h (MCF-7) at 37 °C, filters were fixed and stained with Diff-Quik reagents (Dade Behring, Inc., Newark, DE). The average numbers of six random microscopic fields (x200) was recorded in each experiment.

### Immunofluorescence

Immunofluorescence assays were performed as described previously [23]. Antibodies for E-cadherin (1:100) and HIF-1α (1:50) were obtained from BD Biosciences (San Jose, CA). The cells were examined by confocal microscopy (LSM710; Carl Zeiss, Jena, Germany).

### Chromatin immunoprecipitation

The ChIP assay was performed as described in the protocol from the Millipore ChIP Assay Kit (Upstate Biotechnology, Charlottesville, VA). Antibody for HIF-1α was obtained from Abcam (Cambridge, MA). The promoter-specific primers used were: TWIST1-HRE; forward 5’-GGA CTG GAA AGC GGA AAC TT-3’ and reverse 5’-CGA GGT GTC TGG GAG TTG G-3’, Snail-HRE; forward 5’-GCT GGG CCA GGC TGC TTT GCA-3’ and reverse 5’-GGA CAC CTG ACC TTC CGA CG-3’.

### Subcellular fractionation

The cells were fractionated using the ProteoExtract Subcellular Proteome Extraction Kit (Calbiochem, La Jolla, CA) according to the manufacturer’s instructions. The fractionated samples were analyzed by Immunoblotting.

### Statistics

Data are shown as means ± s.d. Differences between two groups were assessed using the Student’s t-test. Differences among three or more groups were evaluated by analysis of variance, followed by Bonferroni multiple comparison tests.

## Results

### BRMS1 inhibits breast cancer cell EMT

**Fig. 1:**
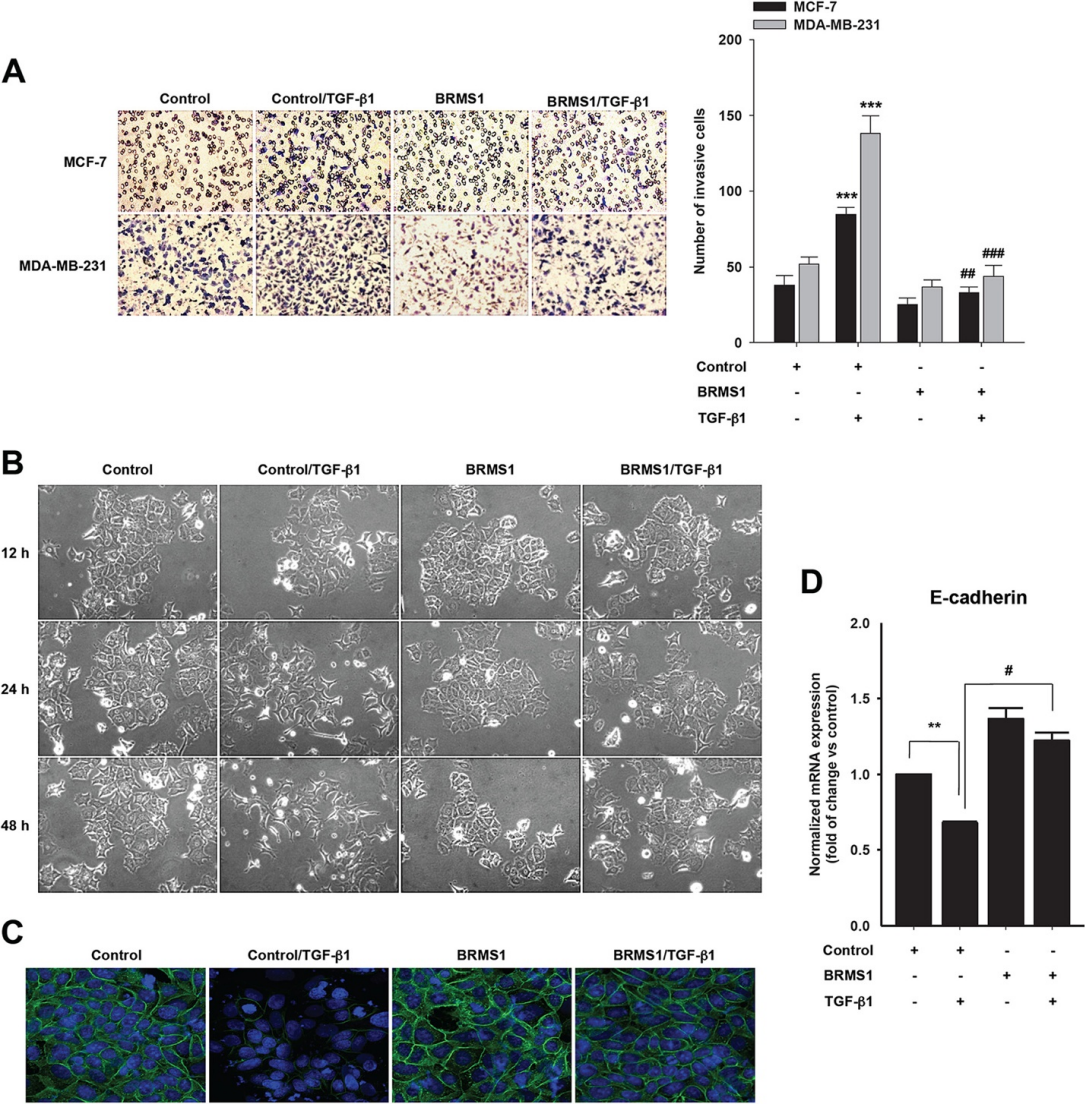
BRMS1 inhibits TGF-β1-induced EMT. **a** The cells were transfected with BRMS1 or empty vector (control), and *in vitro*invasion was analyzed against TGF-β1 (5 ng/ml) (***P < 0.001 *vs* control, ^##^*P* < 0.01 and ^###^*P* < 0.001 *vs* control with TGF-β1). All images original magnification, x200. **b** The MCF-7 cells were transfected with BRMS1 or empty vector (control) and then treated with TGF-β1 (5 ng/ml) for indicated times. The morphology of the cells was examined under light microscope. All images original magnification, x200. **c** The MCF-7 cells were transfected with BRMS1 or empty vector (control) for 48 h and then stimulated with TGF-β1 (5 ng/ml) for 6 h. The cells were fixed and stained to assess expression of E-cadherin (green; E-cadherin, blue; Nuclei). All images original magnification, x200. **d** The MCF-7 cells were transfected with BRMS1 or empty vector (control) for 48 h, followed by stimulation with TGF-β1 (5 ng/ml) for 6 h. The E-cadherin transcript was analyzed by quantitative RT–PCR. Relative mRNA levels normalized to the expression of the housekeeping gene, GAPDH (**P < 0.01 *vs* control, ^#^*P* < 0.05 *vs* control with TGF-β1). Representative results were presented from at least three independent experiments with similar results

Given that BRMS1 has been known to attenuate cancer cell metastasis, we first determined whether BRMS1 inhibits TGF-β1-induced breast cancer cell invasion. Indeed, ectopic expression of BRMS1 significantly inhibited TGF-β1-induced breast cancer cell invasion (Fig. 1a). Since EMT process is important for cancer cell invasion, we next determined whether BRMS1 regulates breast cancer cell EMT. Stimulation of the cells with TGF-β1 efficiently induced morphological change from epithelial to mesenchymal phenotype of breast cancer cells (Fig. 1b). However, overexpression of BRMS1 strongly inhibited TGF-β1-induced EMT. In addition, immunofluorescence analysis showed that BRMS1 restored E-cadherin expression downregulated by TGF-β1 (Fig. 1c). Consistent with these findings, we observed that BRMS1 efficiently inhibited TGF-β1-induced reduction of E-cadherin transcript (Fig. 1d). Therefore, these data strongly suggest that BRMS1 inhibits TGF-β1-induced breast cancer cell EMT.

### BRMS1 inhibits Snail and TWIST1 expression

**Fig. 2:**
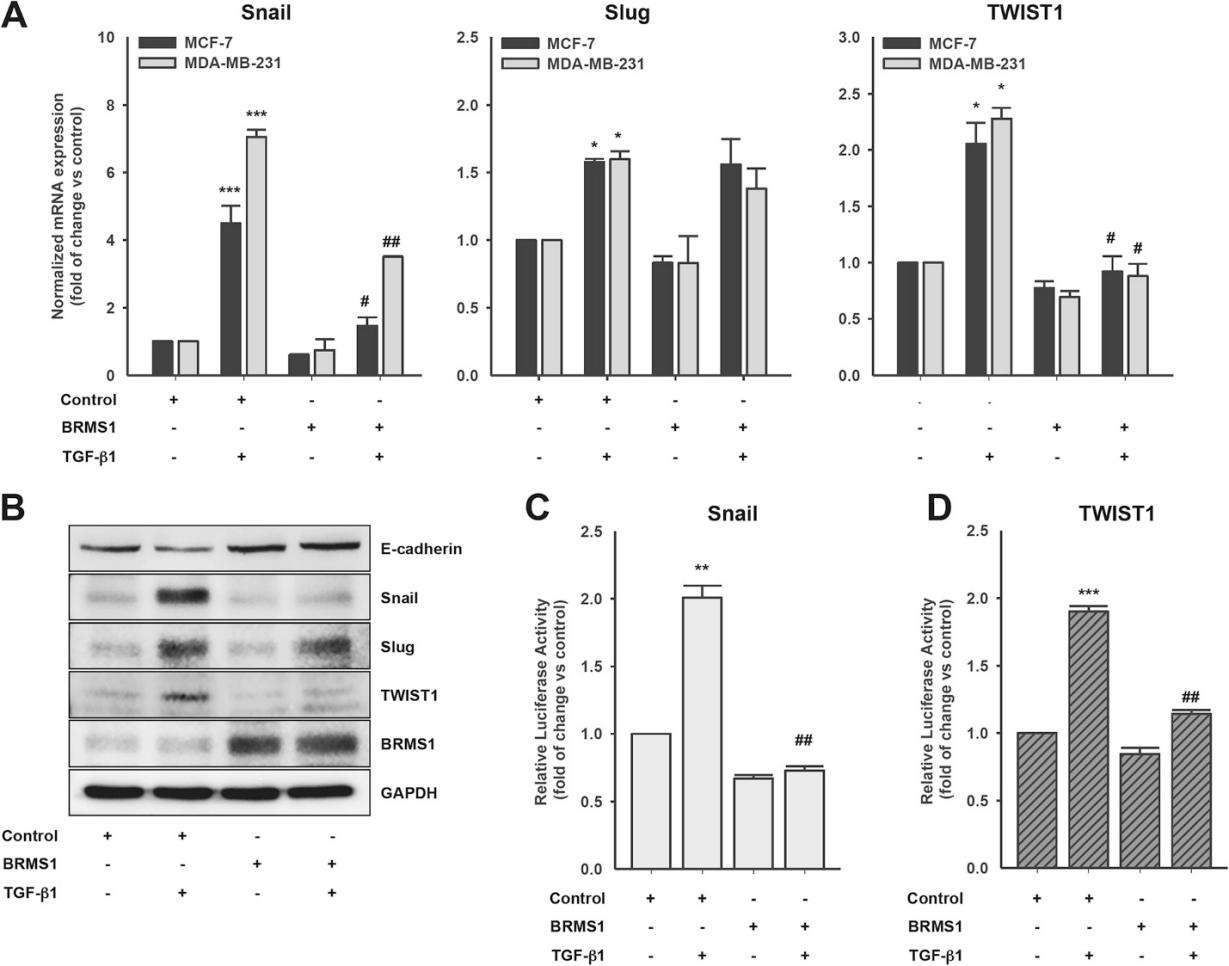
BRMS1 inhibits Snail and TWIST1 expression. **a** The cells were transfected with BRMS1 or empty vector (control) for 48 h and then stimulated with TGF-β1 (5 ng/ml) for 6 h. Quantitative RT–PCR. Relative mRNA levels normalized to the expression of the housekeeping gene, GAPDH (*P < 0.05 and ***P < 0.001 *vs* control, ^#^*P* < 0.05 and ^##^*P* < 0.01 *vs* control with TGF-β1). **b** The MDA-MB-231 cells were transfected with BRMS1 or empty vector (control) for 48 h and then stimulated with TGF-β1 (5 ng/ml) for 6 h. Immunoblotting. **c** and **d** The MDA-MB-231 cells were cotransfected with the empty vector (control), BRMS1 and indicated reporter plasmids for 48 h, followed by stimulation with TGF-β1 (5 ng/ml) for 24 h. Luciferase activity (**P < 0.01 and ***P < 0.001 *vs* control,^##^*P* < 0.01 *vs* control with TGF-β1). Representative results were presented from at least three independent experiments with similar results

We next determined the underlying mechanism by which BRMS1 inhibits TGF-β1-induced breast cancer cell EMT. Stimulation of the cells with TGF-β1 significantly induced mRNA expression of EMT factors Snail, Slug and TWIST1 (Fig. 2a). However, overexpression of BRMS1 markedly inhibited Snail and TWIST1 but not Slug transcript (Fig. 2a). Immunoblotting analysis also showed that BRMS1 decreased TGF-β1-induced Snail and TWIST1 expression (Fig. 2b). In addition, BRMS1 significantly inhibited TGF-β1-induced promoter activities of Snail (Fig. 2c) and TWIST1 (Fig. 2d), confirming that BRMS1 reduces not only TWIST1 but also Snail expression. Therefore, these results strongly suggest that BRMS1 attenuates TGF-β1-induced breast cancer cell EMT through downregulation of both Snail and TWIST1.

### HIF-1α is important for both Snail and TWIST1 expression

**Fig. 3:**
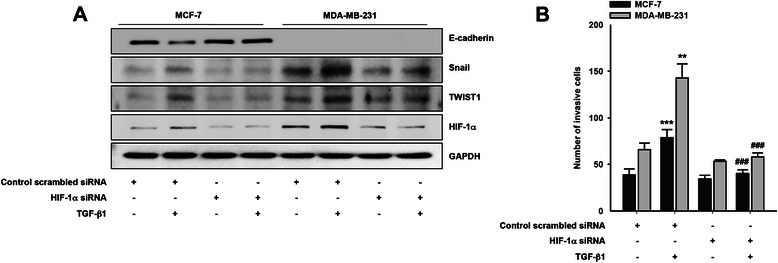
HIF-1α is important for TGF-β1-induced Snail expression. **a** The cells were transfected with indicated siRNAs and then stimulated with or without TGF-β1 (5 ng/ml) for 6 h. Immunoblotting. **b** The cells were transfected with indicated siRNAs and *in vitro* invasion was analyzed against TGF-β1 (5 ng/ml) (**P < 0.01 and ***P < 0.001 *vs* control scrambled siRNA, ^###^*P* < 0.001 *vs* control scrambled siRNA with TGF-β1). Representative results were presented from at least three independent experiments with similar results

Since hypoxia increased Snail [24] and TWIST1 [2] expression, we next determined whether HIF-1α is implicated in TGF-β1-induced Snail and TWIST1expression in breast cancer cells. Stimulation of the cells with TGF-β1 significantly upregulated Snail and TWIST1 expression, while E-cadherin expression was reduced (Fig. 3a). However, silencing HIF-1α showed opposite effects. HIF-1α siRNA reduced both Snail and TWIST1 expression concomitant with increased E-cadherin (Fig. 3a). Consistently, silencing HIF-1α abrogated TGF-β1-induced breast cancer cell invasion (Fig. 3b). Therefore, these data strongly suggest that HIF-1α is important for TGF-β1-induced Snail and TWIST1 expression and cancer cell invasion.

### BRMS1 inhibits HIF-1α expression

**Fig. 4:**
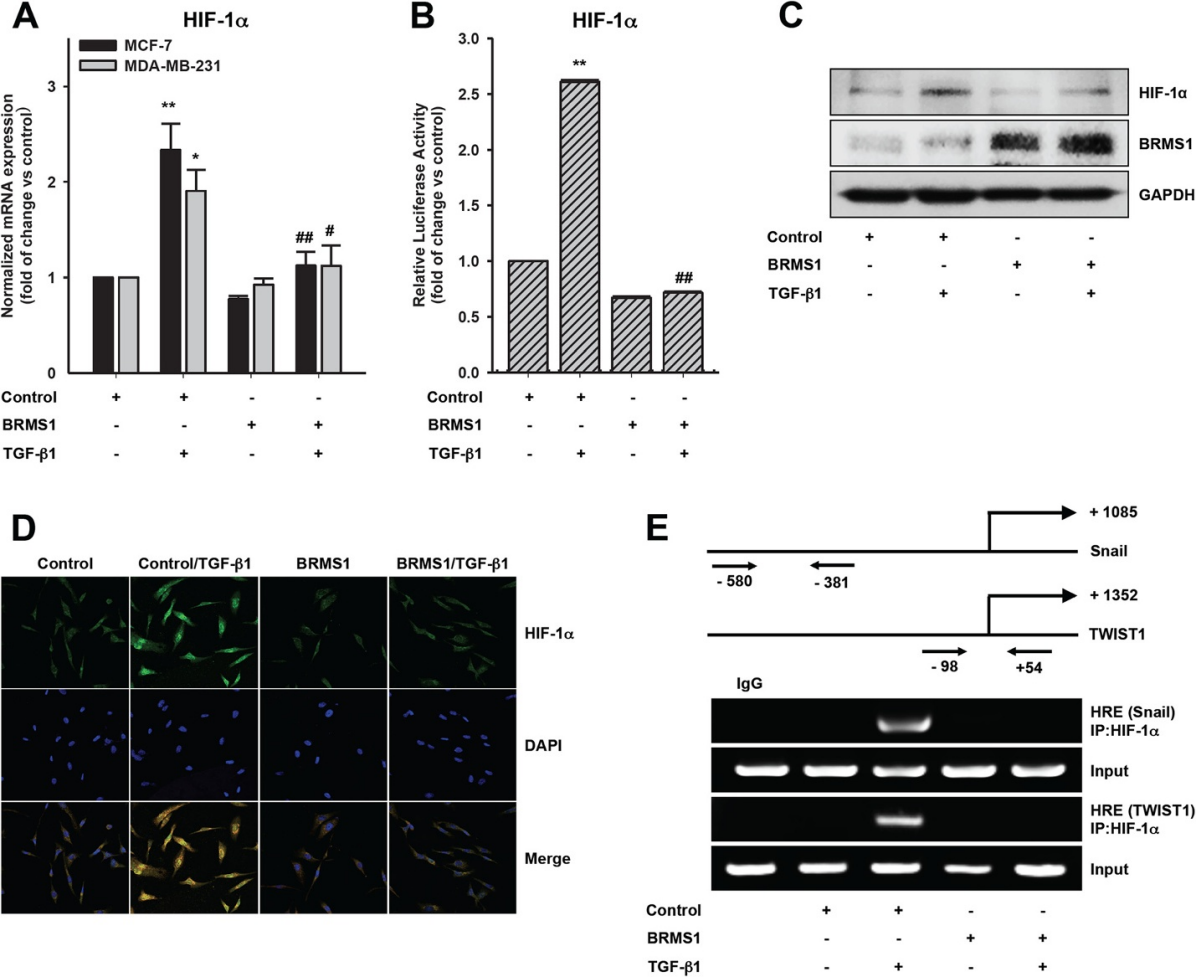
BRMS1 inhibits TGF-β1-induced HIF-1α expression. **a** The cells were transfected with BRMS1 or empty vector (control) for 48 h and then stimulated with TGF-β1 (5 ng/ml) for 6 h. HIF-1α mRNA expression was analyzed by quantitative RT–PCR. Relative HIF-1α mRNA levels normalized to the expression of the housekeeping gene, GAPDH (*P < 0.05 and **P < 0.01 *vs* control, ^#^*P* < 0.05 and ^##^*P* < 0.01 *vs* control with TGF-β1). **b** The MDA-MB-231 cells were co-transfected with the empty vector (control), BRMS1 and HIF-1α reporter plasmids for 48 h, followed by stimulation with TGF-β1 (5 ng/ml) for 24 h. Luciferase activity (**P < 0.01 *vs* control, ^##^*P* < 0.01 *vs* control with TGF-β1). **c** The MDA-MB-231 cells were transfected with BRMS1 or empty vector (control) for 48 h and then stimulated with TGF-β1 (5 ng/ml) for 6 h. Immunoblotting. **d** The MDA-MB-231 cells were sequentially transfected with indicated vectors for 48 h and then stimulated with TGF-β1 (5 ng/ml) for 6 h. The cells were fixed and stained to assess expression of HIF-1α. **e** The MDA-MB-231 cells were transfected with indicated vectors before TGF-β1 (5 ng/ml) treatment. The ChIP assay was performed as described in Materials and Methods. Representative results were presented from at least three independent experiments with similar results

Given that BRMS1 inhibits Snail and TWIST1 expression and that hypoxic condition induces Snail [24] and TWIST1 [2] expression in breast cancer cells, we hypothesized that BRMS1 inhibits HIF-1α expression. Indeed, overexpression of BRMS1 strongly inhibited TGF-β1-induced HIF-1α mRNA expression (Fig. 4a). In addition, ectopic expression of BRMS1 significantly reduced TGF-β1-induced promoter activities of HIF-1α (Fig. 4b). We also observed that BRMS1 strongly reduced TGF-β1-induced HIF-1α expression (Fig. 4c). In addition, immunofluorescence analysis showed that BRMS1 downregulated TGF-β1-induced HIF-1α expression (Fig. 4d). Further, we observed that BRMS1 abrogated TGF-β1-induced binding of HIF-1α on a promoter region of Snail [25] and TWIST1 [26] (Fig. 4e). Together, these results suggest that BRMS1 inhibits TGF-β1-induced HIF-1α expression and subsequent Snail and TWIST1 expression.

### NF-κB is important for HIF-1α expression

**Fig. 5:**
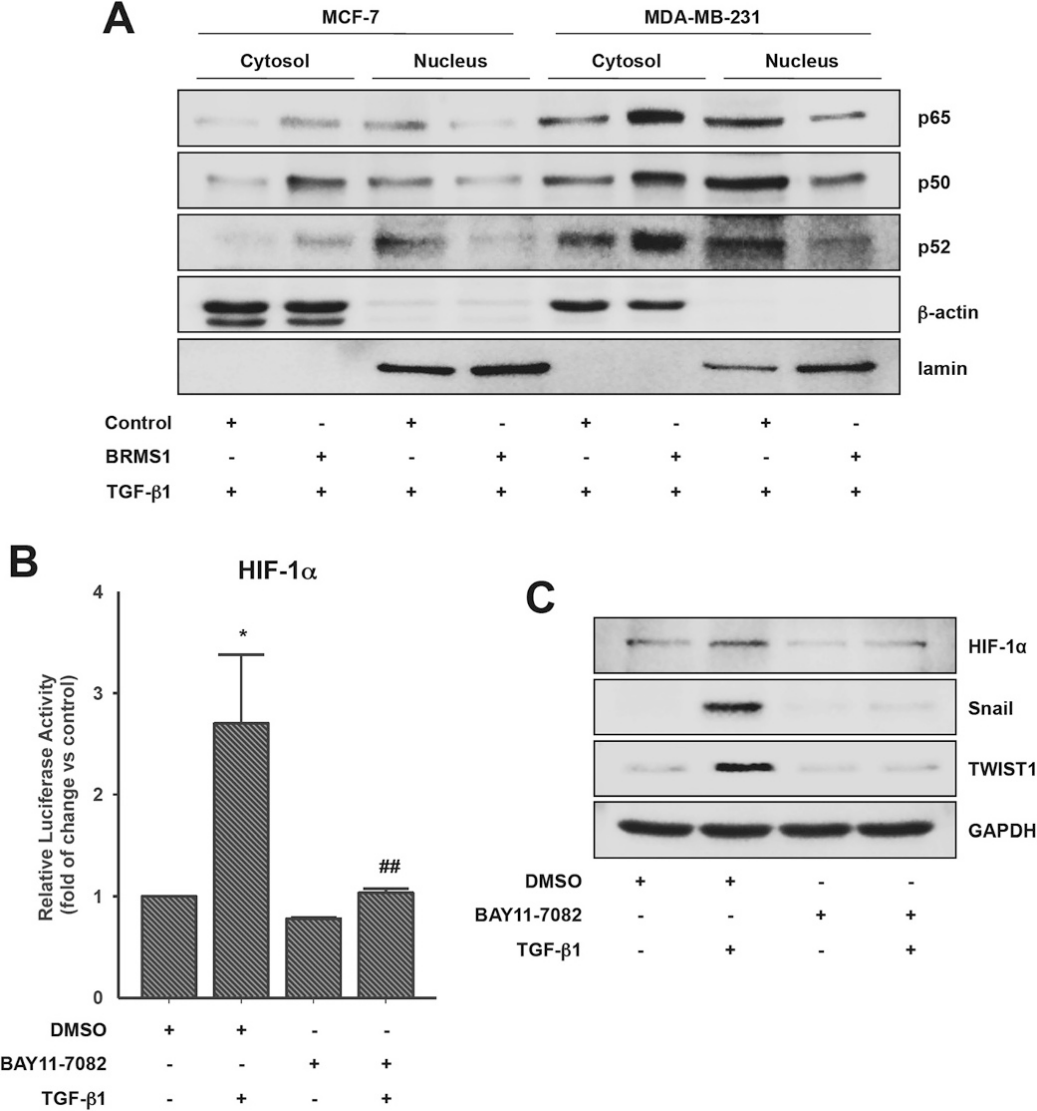
NF-κB is important for HIF-1α expression. **a** The cells were transfected with BRMS1 or empty vector (control) for 48 h and then stimulated with TGF-β1 (5 ng/ml) for 6 h. Immunoblotting. **b** The MDA-MB-231 cells transfected with a luciferase vector containing a HIF-1α promoter were pretreated with BAY11-7082 (5 μM) for 1 h, followed by stimulation with TGF-β1 (5 ng/ml) for 24 h. Luciferase activity (*P < 0.05 *vs* DMSO, ^##^*P* < 0.01 *vs* TGF-β1 treatment only). **c** The serum-starved MDA-MB-231 cells were pretreated with BAY11-7082 (5 μM) for 1 h and then stimulated with TGF-β1 (5 ng/ml) for 6 h. Immunoblotting. Representative results were presented from at least three independent experiments with similar results

Since NF-κB is one of important transcription factors for HIF-1α transcript [27], we next determined whether NF-κB is important for HIF-1α transcript expression. TGF-β1 induced translocation of NF-κB subunits from cytosol to nucleus (Fig. 5a). However, BRMS1 strongly inhibited TGF-β1-induced translocation of NF-κB subunits. In addition, pretreatment of the cells with a pharmacological inhibitor of NF-κB, BAY11-7082 markedly inhibited TGF-β1-induced HIF-1α promoter activity (Fig. 5b). Further, BAY11-7082 abrogated TGF-β1-induced HIF-1α and TWIST1 expression (Fig. 5c). Therefore, these data strongly suggest that NF-κB is implicated in HIF-1α expression and consequent Snail and TWIST1 expression.

## Discussion

In our current study, we elucidate the underlying mechanism by which BRMS1 attenuates breast cancer cell invasion. We demonstrate that both Snail and TWIST1 are important for TGF-β1-induced breast cancer cell invasion. Strikingly, our present data show that BRMS1 downregulates not only TWIST1 but also Snail expression, thereby attenuating TGF-β1-induced breast cancer cell EMT and invasion. Moreover, our data show that HIF-1α mediates TGF-β1-induced Snail and TWIST1 expression and that BRMS1 inactivates NF-κB to reduce HIF-1α transcript, leading to downregulation of TGF-β1-induced Snail and TWIST1 expression. These finding suggest a critical role of HIF-1α through NF-κB activation in TGF-β1-induced Snail and TWIST1 expression and their inhibition by BRMS1 for suppressing breast cancer progression.

BRMS1 has been known to attenuate TWIST1 expression [11, 28]. More recently, Liu *et al*. [16] proposed that BRMS1 suppresses TWIST1 expression and subsequent NSCLC metastasis. In the present study, we provide evidence that in addition to TWIST1, BRMS1 attenuates breast cancer cell invasion through downregulating Snail expression. First, BRMS1 inhibits TGF-β1-induced Snail and TWIST1 but not Slug expression. Second, silencing either Snail or TWIST1 expression recovered TGF-β1-induced E-cadherin expression and breast cancer cell EMT. More importantly, Snail siRNA significantly inhibited TGF-β1-induced breast cancer cell invasion.

HIF-1α induced by hypoxia and growth factors has been shown to mediate EMT and metastasis of various cancer cells. HIF-1α was reported to upregulate TWIST1 expression to induce morphological change of epithelial cells to mesenchymal phenotype [2]. In addition, our recent results suggest that HIF-1α is important for TWIST1 expression and prostate cancer cell invasion [23, 26]. Further, recent study shows that HIF-1α induces histone deacetylase 3 (HDAC3) which in turn cooperate with Snail to induce EMT and metastatic phenotypes [29]. Consistent with these notions, we demonstrated in the present study that TGF-β1 induces HIF-1α expression, which in turn upregulates TWIST1 expression. Notably, we observed that HIF-1α is also implicated in Snail expression in breast cancer cells which was not detected in prostate cancer cells [23], suggesting the differential role of HIF-1α in Snail expression depending on the cellular context. Consistent with this notion, tumor hypoxia correlates with overexpression of HIF-1α, and consequently with TWIST and Snail expression [2]. Since Snail has been known to enhance TWIST1 protein stability in mouse breast epithelial NMuMG cells [30], further study to explore the crosslink between Snail and TWIST1 expression is warranted to determine the underlying mechanism of TGF-β1-induced breast cancer cell EMT and aggressiveness.

## Conclusion

Our results demonstrate that BRMS1 attenuates TGF-β1-induced breast cancer cell invasion through inhibition of NF-κB and subsequent reduction of HIF-1α expression required for Snail and TWIST1 expression, uncovers a new mechanism through which BRMS1 suppresses breast cancer progression.

## Abbreviations

BRMS1: breast cancer metastasis suppressor 1
EMT: epithelial-to-mesenchymal transition
TGF-ß1: transforming growth factor beta 1
EGF: epidermal growth factor
HIF-1α: hypoxia-inducible factor 1alpha
NF-κB: nuclear factor-kappaB
siRNA: Small interfering RNA
ChIP: Chromatin immunoprecipitation
